# Malignant epidural spinal cord compression secondary to testicular cancer (mixed choriocarcinoma and seminoma) in the immediate post radical orchidectomy period

**DOI:** 10.1002/ccr3.2131

**Published:** 2019-04-02

**Authors:** Alain Mwamba Mukendi, Robin Friedman, Abdullah Ismail

**Affiliations:** ^1^ Urology Department, Chris Hani Baragwanath Academic Hospital University of the Witwatersrand Soweto South Africa; ^2^ Anatomical Pathology Department, Chris Hani Baragwanath Academic Hospital University of the Witwatersrand Soweto South Africa

**Keywords:** choriocarcinoma, choriocarcinoma syndrome, mixed germ cell tumor, seminoma, spinal cord compression

## Abstract

Acute spinal cord compression in the immediate postoperative period from a possible choriocarcinoma syndrome (CCS) on hemorrhagic epidural spinal metastasis has never been described before and needs to be promptly recognized and managed. A low hemoglobin associated with choriocarcinoma should raise suspicion of this syndrome.

## 1 INTRODUCTION

Testicular cancer is less common in black males. The highest incidence of these neoplasms worldwide has been reported in white males.^1^ Tumors of the testis constitute a variety of neoplasms with wide‐ranging biological behaviors, histopathological, and clinical findings.^2^ Mixed germ cell tumors include combinations of seminomatous and nonseminomatous histological types, with the most common types being mixed embryonal carcinoma and teratoma, mixed teratoma and seminoma, as well as choriocarcinoma and teratoma.^3^, ^4^ The combination of seminoma and choriocarcinoma is noted to be an exceptionally rare occurrence.^2^

We report the first ever case of this unusual combination associated with spinal cord compression in the immediate post radical orchidectomy period in a black patient from a possible choriocarcinoma syndrome on epidural spinal metastasis. A brief review on pathology and management is herein discussed.

## 2 CASE REPORT

A 27‐year‐old black male was admitted in the medical ward with a 1‐month history of dyspnea and 2 days history of hemoptysis. He also reported a one‐year history of a painless left testicular mass. He had no history of undescended testes nor a known family history of testicular cancer, no backache. On examination, he was dyspneic, with oxygen saturation of 83% on room air for which a facemask oxygen was placed and had a marked gynecomastia. A firm, irregular, and nontender left testicular mass measuring about 10 cm × 7 cm was noted. No neurological deficit was present on admission. He was transferred the following day to the urology ward where he spent less than 24 hours before his urgent referral to the oncology department away from our institution.

**Figure 1:**
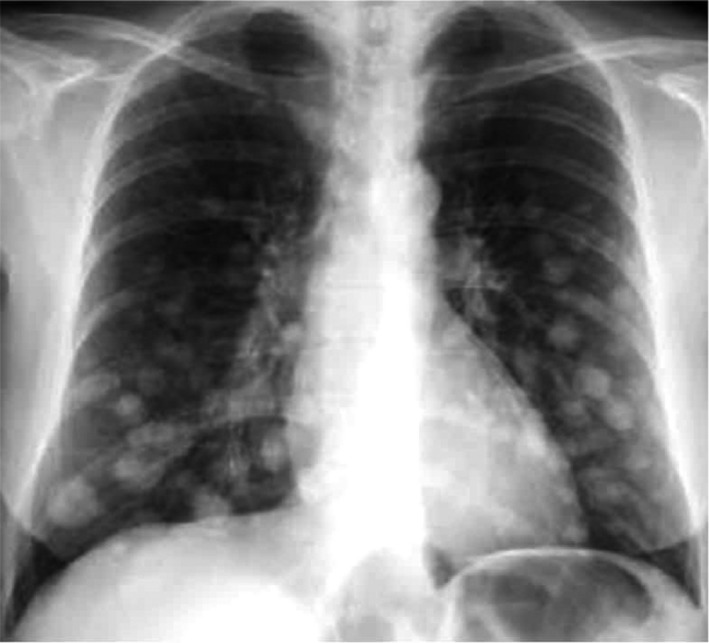
Cannon ball lesions seen on chest X‐ray

Initial investigations showed the following:

Cannon ball lesions on chest X‐ray (Figure 1);
Hemoglobin (Hb) = 4.9 g/dL (normal range = 13.4‐17.5);
Beta‐human chorionic gonadotropin (βhCG) = 807 593 IU/L(normal range = 0);
Alpha‐fetoprotein (AFP) = 2.4 μg/L (normal range = 0.0‐7.0);
Lactate dehydrogenase (LDH) = 1052 U/L(normal range = 48‐115);
Calcium = 1.69 mmol/L (normal range = 2.15‐2.50);
Alkaline phosphatase = 57 U/L(normal range = 53‐128).

**Figure 2:**
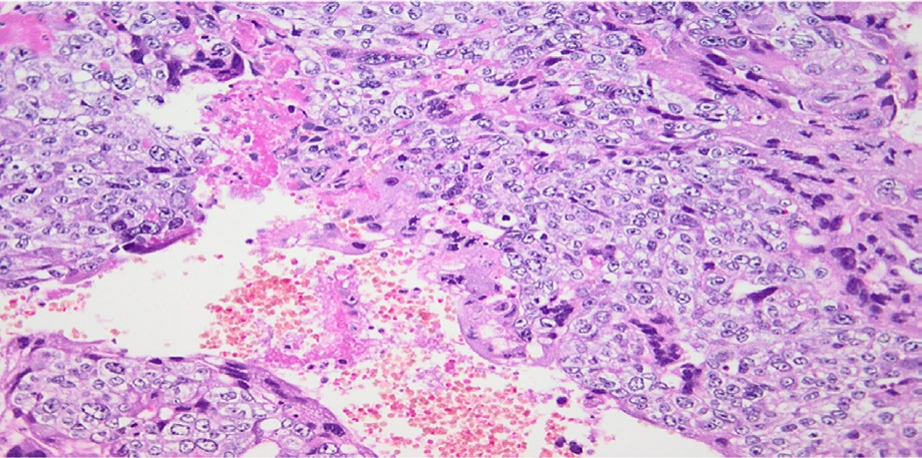
The choriocarcinoma component is composed of an admixture of cytotrophoblasts and multinucleated syncytiotrophoblasts (H&E 200x)

**Figure 3:**
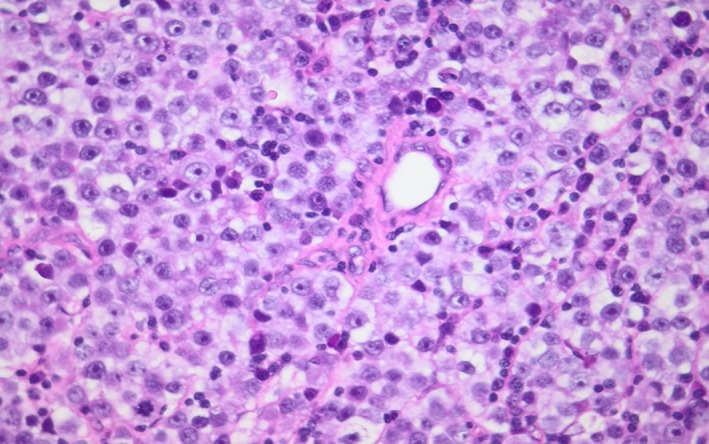
The seminoma component shows diffuse sheets of tumor cells with clear to pale eosinophilic cytoplasm and prominent nucleoli (H&E 400x)

**Figure 4:**
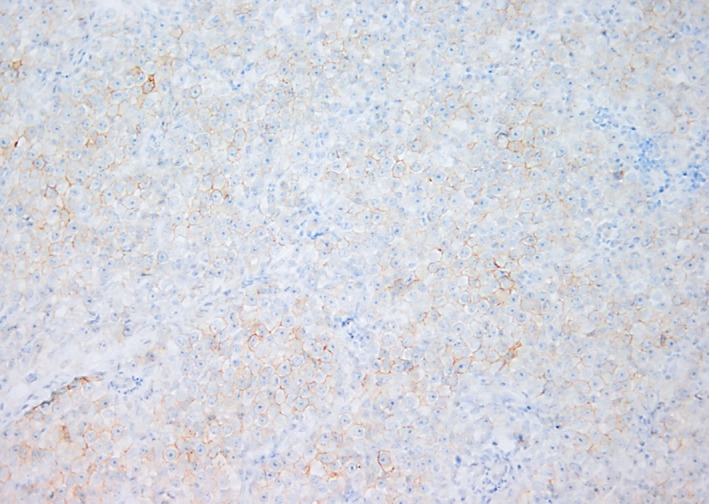
Immunohistochemical staining by CKIT/CD117 highlighting the seminoma component

Histopathology assessment post radical orchidectomy revealed on macroscopic examination the presence of an enlarged left testis that measured 100 × 60 × 55 mm. The cut surface had a nodular appearance with areas of hemorrhage, necrosis, and solid gray tumor nodules. The tunica appeared intact, and the spermatic cord appeared to be uninvolved. Microscopic examination confirmed the presence of a mixed germ cell tumor composed of a choriocarcinoma (90%) and classic seminoma (10%). The choriocarcinoma was composed of cytotrophoblast, intermediate trophoblast, and syncytiotrophoblast (Figure 2). There was extensive hemorrhage and necrosis. The seminomatous component comprised sheets of clear cells which displayed prominent nucleoli (Figure 3). Prominent lymphovascular space invasion was evident. Areas of intratubular germ cell neoplasia were also seen. Immunohistochemical staining performed was CD117 which was in seminoma component of the tumor (Figure 4).

He was transfused to an Hb of 8.2 g/dL prior to surgery. Bronchoscopy could not be performed due to patient's breathing difficulties. Radical orchidectomy was done under spinal anesthesia due to lung metastases. Six hours postoperative, he developed an acute weakness and numbness of both lower limbs after he had recovered from spinal anesthesia. On subsequent examination, both lower limbs had no tone; power: 0/5; absent reflexes bilaterally; no sensation; and a sensory level of T4. He also had a decreased anal tone and loss of bladder control.

**Figure 5:**
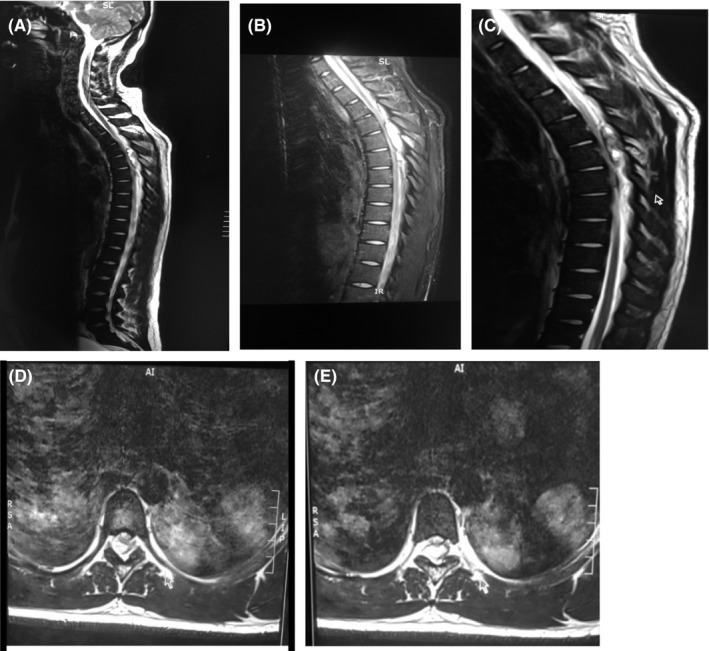
MRI spine sagittal (A, B, C) and axial (D, E) view showing a hyperintense macrolobulated epidural mass with possible intralesional bleed causing spinal cord compression from midvertebral body of T3 to T6

Urgent MRI spine was done and showed a thoracic epidural macrolobulated and heterogeneous mass with predominant T2W hyperintensity causing spinal cord compression from T3 to T6 (Figure 5). MRI findings are also suggestive of a possible intralesional hemorrhage. Postoperative βhCG = 815 815 IU/L. He was sent for urgent radiation and chemotherapy while on corticosteroids. He received eight Gray of emergency radiation therapy to thoracic spine (T2‐T8) and completed one cycle of bleomycin, etoposide, and cisplatin. Patient demised a few days later due to progression of disease.

## 3 DISCUSSION

Testicular neoplasms are very heterogeneous tumors displaying a diversity of histopathological types, clinical courses, and prognosis. Up to 50% of these tumors are classified as mixed germ cell tumors, and several studies have assessed the frequency of the combination of histological components seen in these neoplasms. Choriocarcinoma and seminoma combination is reported to be extremely rare.^2^, ^3^, ^5^, ^6^, ^7^, ^8^ Ashraf mosharafa et al^9^ also reported choriocarcinoma and seminoma to be the less common association.

The seminomatous component is undifferentiated, whereas the choriocarcinoma component is differentiated and secretes βHCG.^10^ Choriocarcinoma is a rare and aggressive tumor that typically manifests with extremely highly elevated serum βHCG levels and disseminated disease. These tumors are typically deemed poor risk at diagnosis because of high serum βHCG level and/or nonpulmonary organ metastases.^11^ Choriocarcinoma commonly spreads by hematogenous routes, and common sites of metastases include lungs, liver, and brain.^11^

It most commonly presents as a component of a mixed germ cell tumor with less than 1% occurring in its pure form. Patients are generally between 20 and 39 years of age and present with gynecomastia, hemoptysis, back pain, neurological and gastrointestinal symptoms due to metastatic spread.

Pulmonary metastatic disease carries the greatest risk with “choriocarcinoma syndrome” used to describe massive hemorrhage at metastatic sites, especially in the lung.^12^ Our patient had hemoptysis and a low hemoglobin. He probably had choriocarcinoma syndrome. Baagar et al^13^ highlighted that hemorrhage can develop from any sites of metastases. Hence, we strongly believe that choriocarcinoma being a very vascular tumor its metastases are very vascular as well and that the sudden neurology deficit in this case may result from a hyperacute hemorrhage within an already vascular epidural metastatic tumor. Hyperacute hemorrhage appears T2W hyperintense on MRI. This may represent another variant of choriocarcinoma syndrome besides the described lung and liver variant. The pathogenesis of this syndrome is unknown. It occurs either spontaneously or few hours after initiation of chemotherapy. The prognosis is poor particularly with βhCG above 50 000 IU/L.^13^

The diagnosis of testicular neoplasm can be made by hematoxylin and eosin stains. If necessary, human chorionic gonadotropin immunohistochemical stains which are positive in the choriocarcinoma component; Octamer‐binding transcription factor 4 (OCT4) and cluster differentiation 117 (CD117) which stain the seminoma component. In cases of diagnostic dilemma, cytokeratin 7 can be of use as it stains trophoblast but not seminoma, embryonal carcinoma, or yolk sac tumors.^12^ When spinal cord compression is suspected, the investigation of choice is magnetic resonance imaging.

The acute management of spinal cord compression consists of corticosteroids, followed by radiation. In patients with poor prognosis, usually a single fraction of eight Gray or 30 Gray in 10 fractions of radiotherapy suffices.^14^ Despite the above treatment, the neurologic deficit tends to persist; therefore, these patients usually require decompression surgery and stabilization. Surgery mainly aims to resect structures bordering the spinal canal dorsally causing compression, secondarily to stabilize the affected segments of the spine. The outcome depends on the extend of spinal metastases, duration of symptoms, and their severity. The lesser the number of metastases; the more acute and less severe the symptoms; the better the outcome.^15^

The management of choriocarcinoma syndrome is quite challenging. It is mostly medical with vigorous support to ensure airway permeability and achieve hemodynamic stability. Surgical excision of the bleeding metastasis may also be an option.^16^

In general, these patients present with a bulky metastatic disease requiring priority chemotherapy and bleomycin, etoposide and cisplatin is the regimen used. The outcome is generally poor regardless of a multidisciplinary treatment approach.^14^, ^17^

## 4 CONCLUSION

Spinal metastases from testicular cancer even though rare are well‐known complications in advanced disease. Spinal cord compression occurring in the immediate postoperative period has never been described before. This is the first reported case of combined choriocarcinoma and seminoma an exceptionally rare histological association with neurologic deficit in the immediate post radical orchidectomy period in a black patient.

In this case, the MRI spine showed epidural spinal cord compression and ruled out any spinal anesthesia related complications (eg, lumbar spine hematoma). The neurologic deficit may have been caused by surgery which may have flared the tumor as evidenced by upsurge in the level of the tumor markers causing possible bleeding from the thoracic epidural metastasis resulting in an acute hematoma, a possible variant of choriocarcinoma syndrome. The real mechanism is not clear at this point.

The outcome was very poor due multiple metastatic sites and progression of disease on treatment (steroids, radiation, and chemotherapy). Even though germ cell tumors are known to be chemosensitive, in a bulky metastatic disease with poor prognostic factors, the response to chemotherapy is generally poor.

## CONFLICT OF INTEREST

None declared.

## AUTHORS CONTRIBUTIONS

AMM: conceived and designed the study, acquired the data, analyzed and interpreted the data, wrote the manuscript, and approved the final manuscript. RF: designed the study, analyzed the data, and approved final manuscript. AI: interpreted the data and wrote a part of the manuscript.

## References

[1] McGlynn KA , Trabert B . Adolescent and adult risk factors for testicular cancer. Nat Rev Urol. 2012;9(6):339‐349. 22508459 10.1038/nrurol.2012.61 PMC4031676

[2] Aneja A , Bhattacharyya S , Mydlo J , Inniss S . Testicular seminomatous mixed germ cell tumor with choriocarcinoma and teratoma with secondary somatic malignancy. J Med Case Rep. 2014;8(1):955. 10.1186/1752-1947-8-1 PMC3917416 24380446

[3] Bahrami A , Ro JY , Ayala AG . An overview of testicular germ cell tumors. Arch Pathol Lab Med. 2007;131(8):1267‐1280. 17683189 10.5858/2007-131-1267-AOOTGC

[4] Williamson SR , Delahunt B , Magi‐Galluzzi C , et al. The World Health Organization 2016 classification of testicular germ cell tumours: a review and update from the International Society of Urological Pathology Testis Consultation Panel. Histopathology. 2016;70(3):335‐346. 27747907 10.1111/his.13102

[5] von Hochstetter AR , Hedinger CE . The differential diagnosis of testicular germ cell tumors in theory and in practice. A critical analysis of two major systems of classification and review of 389 cases. Virchows Arch A Pathol Anat Histol. 1982;396:247. 6291228 10.1007/BF00431386

[6] Krag Jacobsen G , Barlebo H , Olsen J , et al. Testicular germ cell tumours in Denmark 1976–1980: pathology of 1058 consecutive cases. Acta Radiol Oncol. 1984;23:239. 6093440 10.3109/02841868409136019

[7] Mostofi FK , Sesterhenn IA , Davis CJ Jr . Developments in histopathology of testicular germ cell tumors. Semin Urol. 1988;6:171. 2854295

[8] Cheville JC . Classification and pathology of testicular germ cell and sex cord‐stromal tumors. Urol Clin North Am. 1999;26:595‐609. 10494291 10.1016/s0094-0143(05)70201-9

[9] Mosharafa AA , Foster RS , Leibovich BC , et al. Histology in mixed germ cell tumors. Is there a favorite pairing? J Urol. 2004;171:1471‐1473. 15017200 10.1097/01.ju.0000116841.30826.85

[10] Mountzios G , Pavlakis G , Terpos E , et al. Concurrent development of testicular seminoma and choriocarcinoma of the superior mediastinum, presented as cervical mass: a case report and implications about pathogenesis of germ‐cell tumours. BMC Clin Pathol. 2006;6:8. 17112390 10.1186/1472-6890-6-8 PMC1697810

[11] Alvarado‐Cabrero I , Hernández‐Toriz N , Paner GP . Clinicopathologic analysis of choriocarcinoma as a pure or predominant component of germ cell tumor of the testis. Am J Surg Pathol. 2014;38(1):111‐118. 24145647 10.1097/PAS.0b013e3182a2926e

[12] Humphrey PA . Choriocarcinoma of the Testis. J Urol. 2014;192(3):934‐935. 24949806 10.1016/j.juro.2014.06.039

[13] Baagar K , Khan FY , Alkuwari E . Choriocarcinoma syndrome: a case report and a literature review. Case Rep Oncol Med. 2013;2013:697251. 23819084 10.1155/2013/697251 PMC3681292

[14] Cornejo‐Dávilaa V , Santana‐Ríosb ZA , Cantellano‐Orozcoa M, et al. Bone metastases and spinal cord injury secondary to nonseminomatous testicular tumor. Rev Mex Urol. 2014;74(2):99‐103.

[15] Dunning EC , Butler JS , Morris S . Complications in the management of spinal disease. World J Orthop. 2012;3(8):114‐121. 22919567 10.5312/wjo.v3.i8.114 PMC3425630

[16] Motzer RJ , Bosl GJ . A complication of metastatic testicular choriocarcinoma. Urology. 1987;30(2);119‐122. 3617293 10.1016/0090-4295(87)90175-0

[17] Skoch J , Kobylanski K , Rice JeffreyM , Baaj AliA . Metastatic choriocarcinoma to the lumbar spine: Case report and review of literature. Surg Neurol Int. 2014;5:161. 25525554 10.4103/2152-7806.145205 PMC4258720

